# Discovery of Proteins Responsible for Resistance to Three Chemotherapy Drugs in Breast Cancer Cells Using Proteomics and Bioinformatics Analysis

**DOI:** 10.3390/molecules27061762

**Published:** 2022-03-08

**Authors:** Hyo Kyeong Cha, Seongmin Cheon, Hyeyoon Kim, Kyung-Min Lee, Han Suk Ryu, Dohyun Han

**Affiliations:** 1 Transdisciplinary Department of Medicine and Advanced Technology, Seoul National University Hospital, Seoul 03080, Korea; 0619cha@gmail.com (H.K.C.); hyeyoonk@snu.ac.kr (H.K.); 2 Proteomics Core Facility, Biomedical Research Institute, Seoul National University Hospital, Seoul 03080, Korea; s.cheon1995@gmail.com; 3 Center for Medical Innovation, Biomedical Research Institute, Seoul National University Hospital, Seoul 03080, Korea; km601@naver.com; 4 Department of Pathology, Seoul National University Hospital, Seoul 03080, Korea; 5 Department of Pathology, Seoul National University College of Medicine, Seoul 03080, Korea

**Keywords:** proteomics, chemoresistance, breast cancer, prognosis marker, druggable targets

## Abstract

Chemoresistance is a daunting obstacle to the effective treatment of breast cancer patients receiving chemotherapy. Although the mechanism of chemotherapy drug resistance has been explored broadly, the precise mechanism at the proteome level remains unclear. Especially, comparative studies between widely used anticancer drugs in breast cancer are very limited. In this study, we employed proteomics and bioinformatics approaches on chemoresistant breast cancer cell lines to understand the underlying resistance mechanisms that resulted from doxorubicin (DR), paclitaxel (PR), and tamoxifen (TAR). In total, 10,385 proteins were identified and quantified from three TMT 6-plex and one TMT 10-plex experiments. Bioinformatics analysis showed that Notch signaling, immune response, and protein re-localization processes were uniquely associated with DR, PR, and TAR resistance, respectively. In addition, proteomic signatures related to drug resistance were identified as potential targets of many FDA-approved drugs. Furthermore, we identified potential prognostic proteins with significant effects on overall survival. Representatively, PLXNB2 expression was associated with a highly significant increase in risk, and downregulation of ACOX3 was correlated with a worse overall survival rate. Consequently, our study provides new insights into the proteomic aspects of the distinct mechanisms underlying chemoresistance in breast cancer.

## 1. Introduction

Breast cancer accounts for roughly 30% of all cancers in women worldwide and has a 15% death rate; further, incidence rates are increasing at a rate of about 0.5% per year [1]. Breast cancer comprises a heterogeneous group of tumor subtypes, whether defined by the histopathology of the primary tumor, the expression pattern of hormone receptors (estrogen and/or progesterone receptors; ER/PR) and epidermal growth factor receptor 2 (HER2), genetic alterations of transcriptomic traits. These patient-to-patient differences, known as ‘intertumoral heterogeneity’, largely affect patient prognosis and treatment options [2,3,4]. Alongside intertumoral heterogeneity, many studies reported that breast cancers are heterogeneous, with a patient’s primary tumor and individual metastases consisting of many different cells or subclones with different gene expression profiles [2,3,4]. These differences within the tumor are referred to as intratumor heterogeneity, which is caused by a combination of extrinsic factors from the tumor microenvironment and intrinsic parameters including genetic, epigenetic, and transcriptomic traits, the ability of proliferation, migration, and invasion, cell plasticity, and the extent of stemness [2,3,4]. These heterogeneities endow tumors with multiple capabilities and biological characteristics, making them more prone to metastasis, recurrence, and drug resistance [5].

Surgery to remove the tumor and either stage the axillary tumor burden or excise the afflicted axillary lymph nodes are common treatment methods in breast cancer, independent of tumor subtype. Tumor downsizing with systemic therapy before surgery is also recommended for large tumors, and the same systemic therapy is encouraged after surgery. Chemotherapy has generally been regarded as a standard treatment even if the disease is operable [6]. The most commonly used anti-cancer drugs for breast cancer are tamoxifen (Nolvadex), doxorubicin (Adriamycin), and paclitaxel (Taxol) [7,8,9,10]. However, as previously mentioned, chemoresistance that occurs through alteration to drug targets by either innate or acquired abilities has emerged as a major issue that limits the chemotherapy for cancer patients [11].

Chemoresistance of these drugs remains a major cause of therapy failure in breast cancer patients. If we take a more intimate look at them, notably, paclitaxel, a first-line chemotherapy drug for breast cancer, has been reported to develop drug resistance in 90% of patients with breast cancer, particularly metastatic breast cancer [12]. In addition, tamoxifen is an estrogen receptor (ER) antagonist that is commonly used in the treatment of ER-positive breast cancer patients [10]. This resulted in the reduction of the mortality rate by 30% [13]. However, resistance against tamoxifen is still one of the major hurdles in the effective management of breast cancer [14]. Indeed, doxorubicin is an anthracycline antibiotic that is also commonly used to treat cancer. however, its efficiency is hampered by side effects and the development of resistance [15,16,17]. In recent years, it is known that drug resistance in breast cancer is caused by several factors, including host and tumor genetic mutations, epigenetic modifications, and tumor environment [18,19,20]. However, the chemoresistance mechanisms of breast cancer are complicated owing to its heterogeneous nature and have not been fully elucidated.

To overcome these challenges, understanding the proteome mechanisms behind transcriptome profiling from the aspect of treatment can help to improve resistance to cancer therapy. Recent proteomics technologies based on mass spectrometry enable an unbiased investigation of drug-induced changes in protein abundance and post-translational modifications. Several studies on resistance to chemotherapy have recently published data on mass-spectrometry-based chemotherapeutic proteome profiling, which has the potential to discover molecular subtypes and related pathway features that may have been missed in prior transcriptome analyses [21,22,23].

Nevertheless, few studies have performed comprehensive proteomic analysis to elucidate mechanisms of specific drug resistance in breast cancer. In this study, we performed quantitative proteomic analysis to identify proteome differences among doxorubicin-, paclitaxel-, and tamoxifen-resistant breast cancer cells using an isobaric tandem mass tag (TMT) label-based quantitative proteomic approach in combination with comprehensive bioinformatics analysis. By unraveling the protein signatures across tamoxifen, doxorubicin, and paclitaxel and their relationship between drug-resistant cell lines and parent breast cancer cells, our study advances the understanding of the three types of drug resistance and provides potential diagnostic and prognostic markers, as well as testable targets of therapy specific to breast cancer resistant cell subtypes.

## 2. Results

### 2.1. Comparative Proteomic Analysis of Drug-Resistant Breast Cancer Cell Lines

**Figure 1:**
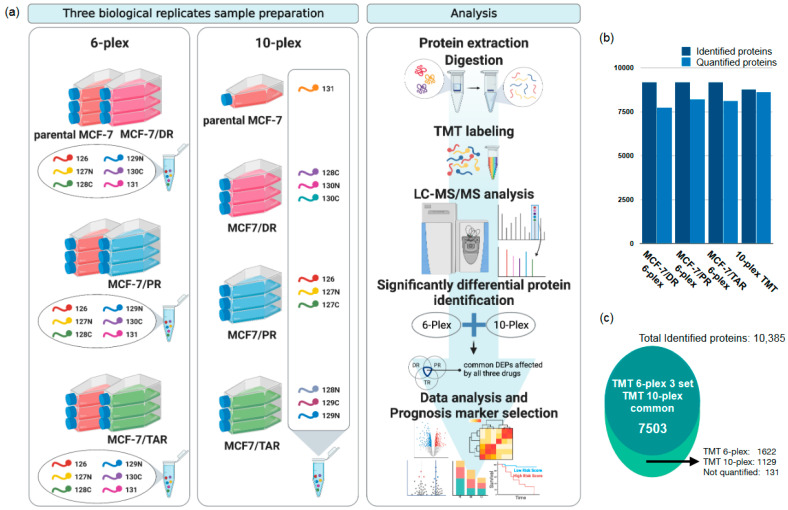
Mass spectrometry-based profiling of drug-resistant breast cancer cell lines. (**a**) Schematic diagram of the proteome analysis of this study. (**b**) A bar plot showing the number of proteins identified and quantitative protein groups per each TMT experiment. (**c**) A total of 10,385 protein groups were identified in our study. Of them, 7503 protein groups were identified and quantified in both TMT 6-plex and TMT 10-plex experiments. The “Not quantified” proteins were identified via search algorithm, but their reporter ions were not detected and were excluded from subsequent quantitative analysis.

We designed a tandem mass tag (TMT) based quantitative proteomic analysis to investigate the global proteome profile of anti-cancer resistance effects of three anticancer drugs, including doxorubicin (DR), paclitaxel (PR), and tamoxifen (TAR). TMT 6-plex was used to compare parental MCF-7 and resistant MCF-7 of each of the three anticancer drugs, and TMT 10-plex was used for direct comparisons between three drug-resistant MCF-7 cells. The experimental procedures for proteomic analysis are illustrated in Figure 1a.

In total, 7756, 8142, and 8225 proteins were identified from the TMT 6-plex data in each of the three drug-resistant cells consisting of MCF-7/DR, MCF-7/PR, and MCF-7/TAR, respectively. A total of 9194 protein groups were observed in at least one of the TMT 6-plex experiments. While the 8633 proteins were identified on the TMT 10-plex data (Figure 1b), 7503 protein groups were commonly quantified in TMT 6-plex and 10-plex experiments (Figure 1c). Approximately, 88% and 86% of the total identified proteins were identified as two or more unique peptides in the TMT 6-plex and TMT 10-plex experiments, respectively. (Figure S1). Although the quantitative variants among multiple channels of labeled proteins showed a suitable reproducibility, we used a non-homologous spiked in the chicken ovalbumin for the internal standard. The coefficient of variation (CV) for ovalbumin was 1.86%, 5.77%, and 5.98% in three TMT 6-plexes, respectively, and 3.46% in the 10-plex. The CV plots according to unique peptides showed that our TMT quantification has good reproducibility and accuracy (Figure S2a,b). All information for identification and quantification was provided in Table S1.

### 2.2. Identification of Protein Expression in Individual Drug-Resistant Cells

**Figure 2:**
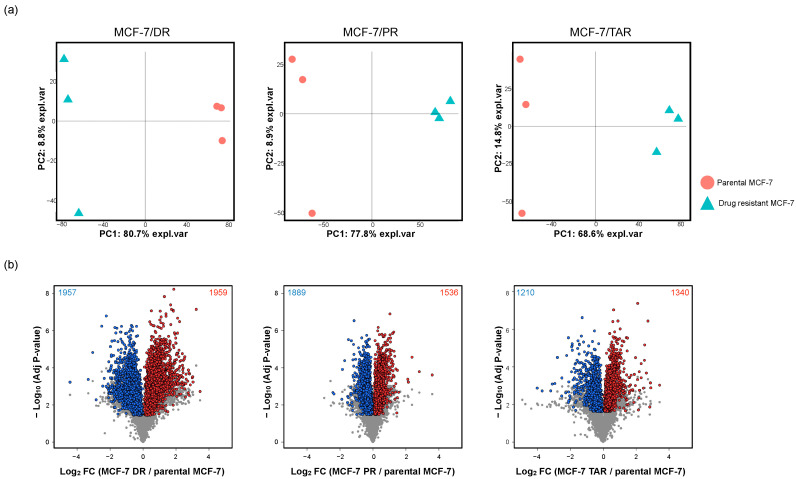
Analysis of differential protein expression with parental MCF-7 and drug-resistant MCF-7 breast cancer cell lines. (**a**) Principal component analysis of proteins quantified in each experimental set. The red spots represent parental MCF-7, and the blue triangle indicates drug-resistant MCF-7 cell lines of each of the three anti-cancer drugs. (**b**) Volcano plots of significantly different protein expression between parental MCF-7 and drug-resistant MCF-7. Volcano plots were drawn using TMT 6-plex data. Significant proteins had an FDR-adjusted *p*-value less than 0.05 for all comparison sets. Additionally, overlapped DEPs between TMT 6-plex and 10-plex datasets were plotted as the red spots (upregulation) and blue spots (downregulation).

To identify significantly different proteins between parental MCF-7 and each of the three drug-resistant MCF-7s from the TMT 6-plex data, pair-wise comparison analysis was performed in MCF-7/DR, MCF-7/PR, and MCF-7/TAR resistant cells compared to parental MCF-7 cells from the TMT 6-plex. First, we performed the principal component analysis (PCA) to compare the proteome profiles among parental MCF-7 cells and drug-resistant MCF-7 cells. Figure 2a shows that the drug-resistant and parental cells are clearly separated, suggesting that there are significant differences in overall proteome expression profiles. T-test analysis showed that 5498, 5349, and 3833 proteins were significantly differentially expressed with adjusted *p*-value < 0.05 between MCF-7/DR, MCF-7/PR, and MCF-7/TAR, respectively, compared to parental MCF-7 cells (Figure S3 and Table S2).

Next, we performed direct comparison analysis among three drugs using TMT 10-plex quantification data. PCA indicated clear separation between three drug resistant breast cancer cells (Figure S4a). Pair-wise comparisons identified 6833, 6694, and 6232 proteins as the differentially expressed proteins (DEPs) in three comparison sets (MCF-7/DR versus MCF-7/PR, MCF-7/DR versus MCF-7/TAR, and MCF-7/PR versus MCF-7/TAR), respectively (Table S2).

Finally, in order to obtain proteins with drug-specific expression alterations as well as protein expression changes associated with the acquisition of drug resistance, DEPs from 6-plex and 10-plex were overlapped (Figure S4b). As a result, 3916, 3425, and 2550 proteins were significantly differentially expressed in each drug resistant cell line compared to the non-resistant cells and to the other two drug-resistant cells (Figure 2b and Figure S4b and Table S3). Due to our goal of elucidating the impact of resistance to three anti-cancer drugs and establishing protein panels with the potential to predict prognosis, these proteins were subjected to further analysis. 

### 2.3. Integrative Analysis of Proteome and Transcriptome from Drug-Resistant Cells

**Figure 3:**
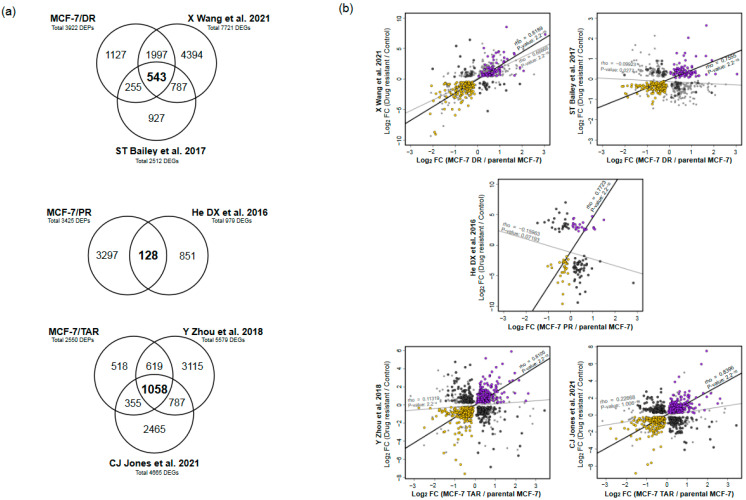
Integrative analysis of proteome and transcriptome of drug-resistant cells. (**a**) Venn diagram of significantly differentially expressed genes between proteome and each publicly available transcriptome. (**b**) Scatter plots of gene-expression correlation between proteome and transcriptome data from each study. Genes for mRNA and protein with increasing concordance were indicated with purple, while genes with decreasing concordance were marked with yellow. Genes that showed discordant changes between proteome and all transcriptome profiles were indicated as dark grey.

Despite the hierarchical organization of gene expression via central dogma, the relationship between transcript and protein expression levels is highly variable in mammalian cells. In order to identify trends related to drug-resistance with high consistency between mRNA and proteins, we compared differential gene expression profiles from publicly available transcriptome profiles for each drug-resistant MCF-7 to the data produced by microarray and RNA sequencing (Figure 3a and Table S4).

**Figure 4:**
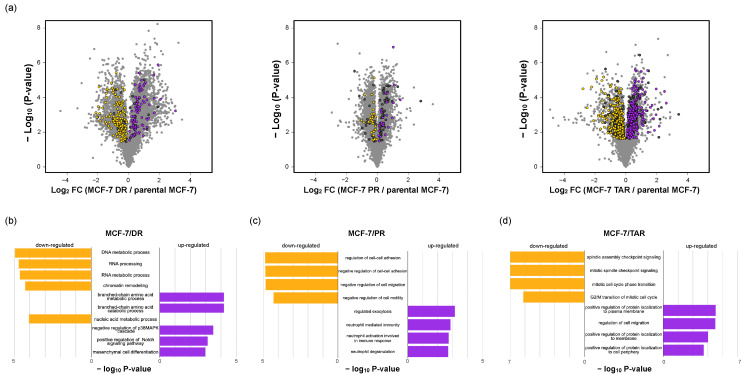
Functional ontology enrichment analysis of genes with concordant changes in proteome and transcriptome. (**a**) Volcano plots of the fold change of protein expression levels. The significantly expressed genes on resistant cells with a positive expression between proteome and transcriptome (purple, upregulated; yellow, downregulated); genes with discordant changes between proteome and transcriptome were labeled as dark grey. (**b**–**d**) Gene ontology analysis results showing biological process enriched by positive expressed genes between proteome and transcriptome (purple, upregulated; yellow, downregulated).

We processed the data from two transcriptome profiles of MCF-7/DR that had been deposited into the public GEO database from independent studies [24,25]. In total, 7721 and 2512 genes were significantly differentially expressed (q-value < 0.05) from independent transcriptome profiling studies of MCF-7/DR, of which 543 genes were detected in three datasets (Figure 3a). Among them, we found 207 genes that had the same direction in both proteome and two sets of transcriptome data (*rho* = 0.81 and 0.76, respectively) (Figure 3b, Figure 4a and Figure S5a). Of the genes with the same direction in a brief positive correlation of gene expression between proteome and transcriptome data, 79 were found to have been upregulated, while 128 genes were downregulated (Figure 3a,b).

Next, we collected processed RNA sequencing data of MCF-7/PR that had been deposited in the public GEO database [26]. A total of 979 genes were significantly differentially expressed (q-value < 0.05) from publicly available RNA sequencing data of MCF-7/PR, of which 128 genes had been detected in our proteome data (Figure 3a). Among them, we found 54 and 74 genes that had the same direction or differential direction on both proteome and transcriptome data, respectively (Figure 3b, Figure 4a and Figure S5a). The genes with the same direction and a positive correlation of gene expression between proteome and transcriptome data were highly correlated, with *rho* = 0.7723 (*p*-value < 2.2 × 10^−11^, and consisted of 23 upregulated genes and 31 downregulated genes in MCF-7/PR cells (Figure 3b, Figure 4a and Figure S5b).

Finally, we processed the publicly available RNA sequencing data of MCF-7/TAR that had been deposited in the NCBI SRA database from two independent studies [27,28]. A total of 5579 and 4665 differentially expressed genes (q-value < 0.05) were identified from independent transcriptome profile study of MCF-7/TAR, with 1058 genes found to be significantly differentially expressed in both proteome and transcriptome data; 415 genes had concordant changes and 300 genes showed a discordant direction between proteome and transcriptome profiles (Figure 3a,b). Compared to the control, 196 genes were upregulated in MCF-7/TAR, whereas 219 genes were downregulated (Figure 3b, Figure 4a and Figure S5c). The genes showed high correlation between proteome and the two transcriptome datasets (*rho* = 0.8104 and 0.8306). 

### 2.4. Functional Analysis of Correlated Expression of Genes between Proteome and Transcriptome in Resistant Cells

We next applied a functional classification analysis to each group of genes defined by concordant directions between proteome and transcriptome from individual drug-resistant cells. Based on the biological process of gene ontology with Enrichr, the genes positively upregulated (*n* = 79) on MCF-7/DR cells and other transcriptome profiles were enriched by the branch-chain amino acid (BCAA) catabolic process (*p*-value < 6.44 × 10^−5^), negative regulation of p38 MAPK cascade (*p*-value < 3.19 × 10^−4^), positive regulation of Notch signaling pathway (*p*-value < 7.47 × 10^−4^), and mesenchymal cell differentiation (*p*-value < 0.001). The majority of proteins with concordant decrease (*n* = 128) are involved in nucleic acid regulation, such as RNA processing (*p*-value < 1.91 × 10^−5^), chromatin remodeling (*p*-value < 5.03 × 10^−5^), and nucleic acid metabolic process (*p*-value < 8.88 × 10^−5^) (Figure 4b and Table S5).

In the case of MCF-7/PR cells, concordantly increasing (*n* = 23) and decreasing (*n* = 31) genes between proteome and transcriptome profiles were subjected to enrichment analysis to the biological process of gene ontology in MCF-7/PR. Differentially overexpressed proteins in both proteome and transcriptome datasets were involved in the neutrophil mediated immunity (*p*-value < 1.45 × 10^−5^) and neutrophil activation involved in immune response (*p*-value < 1.40 × 10^−5^). In addition, the downregulated proteins were associated with negative regulation of the cell motility process (*p*-value < 7.22 × 10^−4^) and negative regulation of cell–cell adhesion (*p*-value < 0.002) (Figure 4c and Table S5).

Finally, genes (*n* = 196) that had concordant increase between mRNA and protein in MCF-7/TAR cells are involved in protein localization control (*p*-value < 6.17 × 10^−5^) and positive regulation of motility (*p*-value < 1.27 × 10^−5^). Moreover, spindle assembly checkpoint signaling (*p*-value < 7.61 × 10^−8^), mitotic cell cycle phase transition (*p*-value < 8.00 × 10^−8^), and G2/M transition of the mitotic cell cycle (*p*-value < 1.71 × 10^−8^) were enriched in genes with concordant decreases (*n* = 219) (Figure 4d and Table S5). 

### 2.5. Analysis of Three Types of Drug-Resistant-Cell-Expressed Proteins and Commonly Regulated Proteins

**Figure 5:**
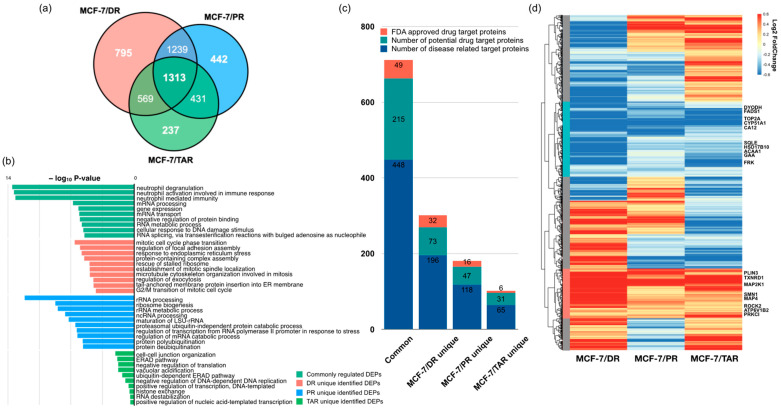
Differentially expressed proteins between the three drug-resistant cell lines. (**a**) Venn diagram showing the overlap of all genes with significantly different expression between parental MCF-7 and drug-resistant cells of Figure 2. (**b**) GO enrichment analysis of DEPs. (**c**) Identification of druggable target proteins on common DEPs and drug-specific DEPs. (**d**) Heatmap using expression levels of the 1313 DEPs commonly significant between parental MCF-7 and each drug-resistant cell line. On the left side of the heatmap, the red bar indicates the 194 upregulated proteins, and the blue bar indicates 295 downregulated proteins. The rest are marked in gray.

To construct a protein panel that can predict anti-cancer drug resistance and prognosis of drug treatment, we overlapped proteins showing drug-specific expression alterations as well as protein expression changes associated with the acquisition of drug resistance. The Venn diagram showed that 795, 442, and 237 proteins specifically related to DR-resistance, PR-resistance, and TAR-resistance, respectively (Figure 5a). Among these proteins, 1313 proteins were common DEPs, indicating that 1313 proteins show altered expressions associated with resistance to the three specific drugs as well as common resistance characteristics compared to non-resistance. 

First, we examined the functional ontology enrichment analysis of common and drug-specific DEPs based on GO annotations (Figure 5b and Table S6). Interestingly, distinct biological processes were enriched in drug-specific proteins and depended on the type of drug. DR-specific proteins were highly associated with mitotic cell cycle phase transition (*p*-value < 2.66 × 10^−7^), regulation of focal adhesion assembly (*p*-value < 1.06 × 10^−6^), response to endoplasmic reticulum stress (*p*-value < 1.57× 10^−6^), and protein-containing complex assembly (*p*-value < 3.05 × 10^−6^). PR-specific proteins were involved in rRNA processing (*p*-value < 7.49 × 10^−13^), ribosome biogenesis (*p*-value < 1.85 × 10^−9^), the rRNA metabolic process (*p*-value < 3.42 × 10^−9^), and ncRNA processing (*p*-value < 2.29 × 10^−8^). Most TAR-specific DEPs were related to cell–cell junction organization (*p*-value < 5.37 × 10^−5^), ERAD pathway (*p*-value < 7.84 × 10^−5^), negative regulation of translation (*p*-value < 9.73 × 10^−5^), and vacuolar acidification (*p*-value < 9.82 × 10^−5^). Next, common DEPs (those that showed significant changes in expression among all three types of drug-resistant cells) were enriched in neutrophil degranulation (*p*-value < 2.99 × 10^−14^), neutrophil-mediated immunity (*p*-value < 6.63 × 10^−14^), mRNA processing (*p*-value < 1.72 × 10^−7^), and cellular response to DNA damage stimulus (*p*-value < 2.36 × 10^−6^).

To discover novel drug target proteins that may respond to drug resistance in breast cancer, we compared these DEPs to three categories of druggable target proteins from the human protein atlas [29]. Comparative analysis showed that the most abundant drug target of disease-related proteins on commonly regulated genes had calculated enrichment scores of 1.6. These results imply that 1313 common DEPs may be involved in the regulation of other drug target genes during development of resistance (Figure 5c). 

Of common DEPs (1313 proteins), TMT 6-plex data indicated that 194 proteins were consistently upregulated in all three drug-resistant MCF-7 cells, and 295 proteins were downregulated (Figure 5d). Especially, we found eight DEPs consistently upregulated in any drug-resistant cell—ATP6V1B2, MAP2K1, MAP4, PLIN3, PRKCI, ROCK2, SMN1, and TXNRD1, which were overexpressed more than 1.2-fold compared with parental MCF-7 cell lines—whereas FADS1, FRK, GAA, HSD17B10, CYP51A1, DHODH, CA12, ACAA1, SQLE, and TOP2A were consistently downregulated (Figure 5d, Table 1 and Table S7).

### 2.6. Discovery of Candidate Anti-Cancer-Drug-Resistant Marker with TCGA and Metabric Survival Data

To assess contribution of drug-resistance-related proteins to breast cancer patient survival, we calculated the overall survival rate of the 194 upregulated and 294 downregulated proteins found in all three drug-resistant cells using survival information of TCGA-BRCA (*n* = 940) and Metabric cohort (*n* = 1468) [30,31]. 

**Figure 6:**
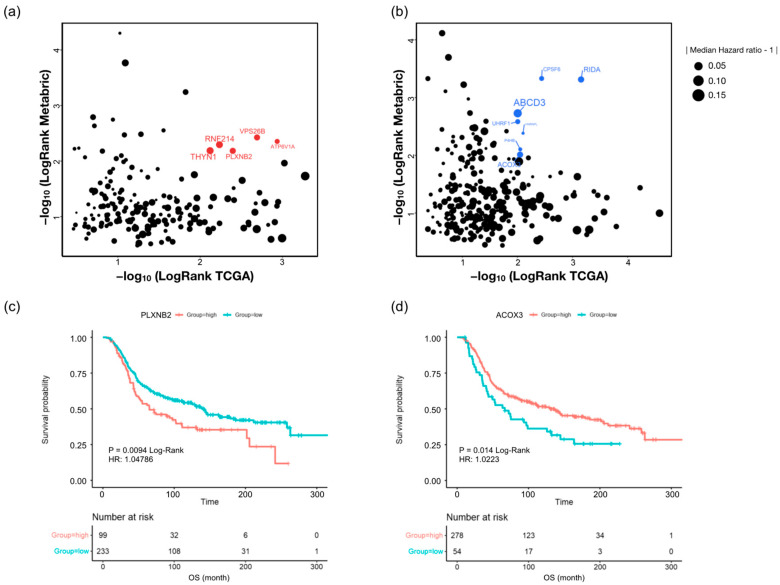
Relationships between drug-resistance-related proteins and overall survival (OS) in breast cancer patients. The log-rank test of overall survival rate of 194 upregulated proteins (**a**) and 294 downregulated proteins (**b**) in TCGA-BRCA pan-cancer (*x*-axis) and Metabric cohorts (*y*-axis). The highly significant log-rank *p*-value in both cohorts was indicated with red (**a**) and blue (**b**). Log-rank analysis of overall survival according to expression level of PLXNB2 (**c**) and ACOX3 (**d**) in patients treated with chemotherapy from Metabric.

Among all 488 proteins, 12 proteins were closely associated with overall survival (OS) in both databases, as determined by Kaplan–Meier (KM) analysis (Figure 6a,b). In upregulated proteins (Table 2), ATP6V1A, VPS26B, PLXNB2, RNF214, and THYN1 are highly associated with shorter OS in both patient populations (log-rank < 0.01) (Figure S6). In addition, downregulation of RIDA, CPSF6, ABCD3, UHRF1, HNRNPL, P4HB, and ACOX3 was associated with shorter OS in both databases (Figure S7). Interestingly, in subgroups of patients treated with chemotherapy (332 patients in Metabric), we found that high plexin-B2 (PLXNB2) expression was associated with shorter OS, whereas downregulation of acyl-coenzyme A oxidase 3 (ACOX3) predicted shorter OS in patients treated with chemotherapy (Figure 6c,d). Significantly, MS/MS spectrums showed that PLXNB2 and ACOX3 are identified with high confidence in our data (Figures S8 and S9). Moreover, expression patterns of these two proteins from TMT 10-plex data suggested that PLXNB2 and ACOX3 are putative prognosis markers of chemotherapy-resistance regardless of drug type (Figure S10).

## 3. Discussion

Here, we performed, for the first time, a comparative proteomic analysis between three drugs for breast cancer chemotherapy. The novelty of our research deals with the application of in-depth quantitative proteomic methodologies to discover the relationship of doxorubicin-, paclitaxel-, and tamoxifen-resistance mechanisms in breast cancer cells. TMT-based in-depth proteome analysis paves the way to a better understanding of underlying molecular mechanisms of drug resistance and helps to identify potential drug targets for breast cancer treatment.

Many studies reported that the relationship between transcript and protein expression levels is highly variable in mammalian cells [32]. The proteome is characterized by large protein-abundance differences, cell-type and time-dependent expression patterns, and post-translational modifications, all of which carry biological information that is inaccessible by genomics or transcriptomics. In our study, we used differential gene expression profiles from publicly available transcriptome profiles of each drug-resistant MCF-7 compared to data produced by microarray and RNA sequencing to identify trends related to drug resistance with high consistency between mRNA and proteins. The overlapped genes with concordant expression patterns between DEPs in our data and differentially expressed genes (DEGs) in public transcriptomics data are highly correlated. However, correlation analysis using overlapping genes, regardless of expression direction, differed depending on the type of drug. Although one dataset for MCF-7/DR from X Wang et al. and two transcriptomics datasets for MCF-7/TAR were significantly positively correlated, data from ST bailey et al. and He Dx et al. showed negative correlation. This is probably because the data from ST bailey et al. data is micro-array data, and the data from He DX et al. is low-depth sequenced RNA-seq data with only one experimental sample size. Therefore, genes with high consistency between mRNA and protein were selected and subjected to further analysis.

Since the 1970s, doxorubicin (DR), an anthracycline antibiotic, has been regarded as one of the most effective treatments for breast cancer [33]. Anthracyclines have been the standard backbone of chemotherapy for breast cancer cure for over three decades [34]. The mechanism of the antineoplastic effect of doxorubicin at the cellular level is drug binding to DNA by insertion between base pairs and inhibition of RNA synthesis by template disorder and steric hindrance [35]. This leads to cell-cycle arrest and subsequent induction of DNA damage related to the apoptotic pathway. 

However, DR’s efficiency is impeded by resistance via several mechanisms. A range of factors contributing to the acquired phenotype of DR resistance in breast cancer have been proposed, including the activation of the nuclear factor erythroid 2-related factor 2 (Nrf2) signaling pathway [36] and the mitogen-activated protein kinase (MAPK)/extracellular-signal-regulated kinase (ERK) [37]. 

In our study, proteins involved in branched-chain amino acid (BCAA) catabolic processes were uniquely upregulated in MCF-7/DR cells. Recent studies have demonstrated that catabolism of BCAAs produces intermediates that are vital for driving triple-negative breast cancer (TNBC) growth and survival [38]. Moreover, BCAA catabolism dysregulation is significantly related to DR chemosensitivity and chemoresistance [39]. In addition, several upregulated proteins were involved in negative regulation of the p38-MAPK cascade. A recent study reported that p38 MAPK inhibitor significantly increases gastric cancer cell sensitivity to doxorubicin [40], which contradicts our enrichment result. Interestingly, that the recombinant, dual-target MDM2/MDMX inhibitor could reverse doxorubicin resistance via the activation of the TAB1/TAK1/p38 MAPK cascade in breast cancer cells [41] is consistent with our result.

Positive regulation of the Notch signaling pathway was also associated with upregulated proteins in MCF-7/DR cells. The Notch signaling pathway played a key role in breast cancer tumorigenesis and progression, as well as therapy resistance and disease relapse in breast cancer patients [42]. Li et al. [43] showed that the inhibition of the Notch-1 signaling pathway with γ-secretase inhibitor could enhance the sensitivity to doxorubicin treatment in MDA-MB-231 cells. Another recent study demonstrated that antibody-specific inhibition of JAG1 sensitizes chemoresistance of TNBC cells in vivo in mice, showcasing an important role for JAG1 and the Notch pathway in promoting chemoresistance in breast cancer [44]. These studies suggest that upregulated proteins involved in positive regulation of the Notch signaling pathway can be potential therapeutic targets for chemoresistance in breast cancer. 

On the other hand, downregulated proteins were mainly involved in chromatic remodeling, including DNA and RNA metabolic process and histone modifications. Interestingly, a recent epigenetic study demonstrated that major histone-modifying enzymes, such as HDAC2, EZH2, and PRMT5, are significantly downregulated in doxorubicin-resistant MCF7 cells [25], suggesting that downregulation or loss of certain regulators in chromatic remodeling may also play an important role in promoting the development of cancer drug resistance.

Paclitaxel (PR) is one of the active chemotherapeutic drugs commonly used to treat metastatic breast cancer [8,9]. Paclitaxel, a class of taxanes, is an anti-tumor drug that binds to β-tubulin and prevents mitosis through microtubule hyperstabilization [9]. Several mechanisms have been reported to understand paclitaxel resistance in breast cancer. Previous studies reported that PR resistance is mediated by the Hippo–LATS signaling pathway and its downstream transcriptional coactivator [45] and overexpression of multidrug transporter genes such as ATP binding cassette subfamily B member 1 (ABCB1, MDR1) and the ATP binding cassette subfamily C member 1 (ABCC1, MRP1) [46]. The additional mechanism described suggested that paclitaxel resistance is caused by a point mutation at β292 (Gln to Glu), β173 (Pro to Ala), and β422 (Tyr to Tyr/Cys) in the β-tubulin gene at the paclitaxel binding site [47,48]. These point mutations are located around the M-loop, nucleotide-binding site, and C-terminus, which are responsible for stabilizing lateral connections between protofilaments, GTP hydrolysis, and MAP binding, respectively. Moreover, changing tubulin isotype expression levels have been linked to the emergence of paclitaxel resistance [49,50]. 

Interestingly, we identified that upregulated proteins in MCF-7/PR are mainly involved in neutrophil activation involved in immune response. Tumor-associated neutrophils (TANs) have been shown to promote tumor progression through a variety of mechanisms, including stimulation of angiogenesis, invasiveness, and releasing growth factors [51,52]. Indeed, tumors are thought to unintentionally stimulate tumor progression by secreting factors that induce wound healing responses from TAN and tumor-associated macrophages [53]. Even neutrophil extracellular traps generated during inflammation may also promote the reawakening of dormant tumor cells [54]. These results suggest an important role of interactions between breast cancer cells and TANs in regulating pro-tumor characteristics in neutrophils and their modulation by therapy resistance [55].

Meanwhile, downregulated proteins in MCF-7/PR were uniquely enriched to cause negative regulation of cell motility and migration. Because chemoresistance can be driven by the motility of the cancer cells within the chemotherapy drug gradient [56], downregulation of proteins that acted as negative regulators of cell motility could induce the migration of breast cancer cells. Especially, the upregulated group contained CLDN3, CDH1, and PTPRK, which have major roles in epithelial–mesenchymal transition (EMT). Recent studies reported that drug-resistant breast cancer cells acquire EMT characteristics and have increased motility and invasion activities by suppression of CLDN3 [57], CDH1 [58], and PTPRK [59]. Moreover, PTPRK is proposed as an important regulator of EMT plasticity in breast cancer [59]. Therefore, the development of plasticity inhibitors may have great potential in cancer treatment, despite limited evidence from clinical studies [60]. 

Tamoxifen is a competitive inhibitor of estrogen action and a hormone-based anti-cancer drug that blocks the binding of estradiol to the ER through positive hormone receptors in cancer cells [61,62]. Tamoxifen is approved as the first-line treatment for the prevention of high-risk breast cancer and is used to treat breast cancer, including delaying recurrence and progression [62,63,64]. However, about 20–30% of tumors are resistant to tamoxifen therapy either prior to treatment or during treatment. Several factors suggested to be responsible for tamoxifen resistance include crosstalk between ER and the growth factor receptor (GFR) network [65], downregulation of ER [14], upregulation of specific GFR [66], activation of PI3/AKT/mTOR pathway [67], PTEN inactivation [68], and induction of NF-κB signaling [69].

Compared with other drug-resistant cells, proteins involved in protein localization control and regulation of cell migration were mainly upregulated in tamoxifen-resistant cells. As expected, our data showed that the molecular mechanisms of tamoxifen resistance might be mainly related to membrane structures. Moreover, previous studies have shown that regulation of protein localization changes is associated with EGFR/ERK and EGFR/AKT signaling activation in tamoxifen-resistant breast cancer cells, indicating that this may be a potential target for enhancing chemosensitivity of breast cancer patients [70]. 

In addition, cell cycle related terms, including spindle assembly checkpoint signaling, mitotic cell cycle phase transition, and G2/M transition of the mitotic cell cycle were significantly enriched in downregulated proteins. Several studies reported that the expression and activity of cell cycle regulators are significantly associated with tamoxifen sensitivity and resistance [71]. Interestingly, our enrichment results showed several proteins involved in cell cycle machinery are downregulated in MCF-7/TAR cells, which is in contrast to the previous studies. Although enrichment analysis is performed using proteins with concordant abundance changes between proteome and transcriptome, discrepancies with previous studies should be addressed in further studies.

Mass-spectrometry-based proteomics is a well-established tool in drug target discovery. Large scale quantification data with protein expression levels and changes of protein abundance makes proteomics particularly valuable in drug target discovery [72]. In this study, we discovered a putative drug target protein in DEPs in all three drug-resistant MCF-7 lines by using the human proteome atlas database containing the druggable target protein candidates. Regardless of whether drug-specific DEPs or common DEPs, a considerable number of druggable target proteins were identified. Among them, common DEPs had the most abundant druggable target proteins. It is important that the large number of proteins changed by all three of the chemoresistant cell lines are included as protein targets for other drugs. These proteins can be presented as potentially druggable target proteins for anti-cancer-drug resistance, further if it is a protein that is already being studied as a target for other drugs and has great potential for novel drug development. 

Finally, we analyzed the prognostic role of common DEPs in three drug-resistant breast cancer cells using public clinical information (TCGA-BRCA and Metabric cohorts). Interestingly, survival analysis in subpopulations of patients treated with chemotherapy suggested that high PLXNB2 expression and low ACOX3 expression were associated with a highly significant increase in risk. 

PLXNB2 is the functional cell surface receptor of ANG, which was originally identified as a tumor angiogenic factor [73,74]. PLXNB2 can also finetune the invasive growth process under both physiological conditions and tumor growth and metastasis [75]. Moreover, overexpression of PLXNB2 proteins is correlated with significantly reduced median survival rate in prostate cancer, glioma, and breast cancer [73]. As PLXNB2 responds to cell proliferation and stress [73], it is expected to show worse drug resistance as it increases. ACOX3, an acyl-CoA oxidase, is known to be involved in peroxisomal branched-chain fatty acid β-oxidation. Although ACOX3 is highly expressed in human prostate cancer tissue compared with paired normal tissues, very low levels of expression are shown in other organs [76,77]. Interestingly, the prognostic role of ACOX3 in breast cancer as well as other cancers is unclear. On the other hand, ACOX2, known to be related to ACOX3, is proposed as a promising prognostic marker in hepatocellular carcinoma [78] and breast carcinomas [79]. This suggests that ACOX3 can be a potential prognostic marker in breast cancer and drug resistance, although prognostic performance of ACOX3 should be confirmed in future experiments. In our data, PLXNB2 and ACOX3 are up- and downregulated, respectively, in all three drug-resistant cells (Table 2). Considering our results and previous studies, PLXNB2 and ACOX3 are proposed as universal prognostic markers of breast cancer associated with chemotherapy resistance.

Our analysis revealed novel properties for the chemical resistance of the three anti-cancer drugs and possible drug targets that could overcome them. The major limitation is that cells were not cotreated with three anti-cancer drugs. Furthermore, additional evaluations for potential clinical applicability will necessitate experimental validation of these results. Ongoing research to correlate combined treatment of the three anti-cancer drugs and clinical responses will address this issue.

## 4. Materials and Methods

### 4.1. Cell Culture

The ER-positive human breast cancer cell line, MCF-7, was obtained from ATCC (Manassas, VA, USA). The tamoxifen (TAR)-, paclitaxel (PR)-, and doxorubicin (DR)-resistant, ER-positive human breast cancer cell lines were kindly provided by Professor Woo Kyung Moon (Department of Radiology, Seoul National University Hospital, Seoul, Korea). All these cell lines were cultured in Dulbecco’s Modified Eagle’s Media (DMEM) (WelGENE, Daegu, Korea) containing 10% fetal bovine serum, 100 units/mL penicillin, and 100 μg/mL streptomycin. MCF-7/TAR cells were cultured in a medium supplemented with 3 μmol/L TAR (Sigma, St. Louis, MO, USA). All cells were incubated at 37 °C in a humidified atmosphere of 95% air/5% CO_2_.

### 4.2. Cell Lysis and Protein Digestion

Cell pellets were lysed with lysis buffer (4% SDS and 2 mM TCEP in 0.1 M Tris pH 8.5). Protein concentration was measured by a BCA-reducing compatible kit (Thermo Fisher Scientific, Waltham, MA, USA). Protein digestion was performed using a filter-aided sample preparation (FASP) procedure as described previously [80,81]. After 200 μg of proteins was precipitated overnight at −20 °C using ice-cold acetone, protein digestion was performed via the two-step FASP procedure as described with some modifications [80,81]. Protein pellets were dissolved in SDT buffer (4% SDS, 10 mM TCEP, and 50 mM CAA in 0.1 M Tris pH 8.0) and loaded onto a 30 K Amicon filter (Millipore, Jaffrey, NH, USA). The buffer exchanges were performed with UA solution (8 M urea in 0.1 M Tris pH 8.5) via centrifugation at 14,000× *g* for 15 min. Following the exchange of buffer with 50 mM TEAB, protein digestion was performed at 37 °C overnight using a trypsin/Lys-C mixture (Promega, Madison, WI, USA) at a 100:1 protein-to-protease ratio. The digested peptides were collected by centrifugation. After the filter units were washed with 40 mM ABC, the second digestion was performed at 37 °C for 2 h using trypsin (enzyme-to-substrate ratio (*w*/*w*) of 1:1000). The peptide concentration was measured by tryptophan assay [82].

### 4.3. TMT Labeling

Tandem mass tag (TMT) labeling was performed according to the manufacturer’s protocol with some modifications. Briefly, TMT 10-plex (Thermo Fisher Scientific, Waltham, MA, USA) or TMT 6-plex reagent (0.8 mg) was dissolved in 100% can. Each 40-µg sample was spiked with 260 ng of peptides derived from ovalbumin for use as an internal standard, ACN was added to the reagent to give a final concentration of 30% (*v/v*). After incubation at room temperature for 1 h, the reaction was quenched with 5% hydroxylamine. The TMT-labeled peptides were pooled at equal concentrations, and the mixtures were dried in a speed vacuum.

### 4.4. High-pH Peptide Fractionation

The TMT-labeled tryptic peptides were fractionated offline using the reversed-phase high-pH strategy as described previously [83]. Before high-pH fractionation, the pooled peptides were desalted using Oasis solid-phase extraction (SPE) columns (Waters, Milford, MA, USA), and the resulting peptides were subjected to Agilent 1290 bioinert HPLC (Agilent, Santa Clara, CA, USA) equipped with an Agilent Zorbax Extend-C18 5 μm 4.6 × 250 mm column. For peptide separation, mobile phase A was 15 mM ammonium hydroxide in water (pH = 10), and mobile phase B was 15 mM ammonium hydroxide in acetonitrile (pH = 10). Ammonium hydroxide was used as the only additive to the mobile phases. The peptides were fractionated with a gradient from 5 to 35% ACN at a flow rate of 0.2 mL/min. A total of 96 fractions were concatenated into 24 fractions and evaporated in a speed vacuum.

### 4.5. Mass Spectrometry and Proteomic Data Analysis

The fractionated peptides were analyzed with a Quadrupole Orbitrap mass spectrometry (Q-exactive plus, Thermo Fisher Scientific, Waltham, MA, USA) equipped with an Ultimate 3000 RSLC system (Dionex, Sunnyvale, CA, USA) via a nanoelectrospray source. The peptides were separated on the two-column system with a trap column (300 μm I.D. × 5 mm, C18 3 μm, 100 Å) and an analytical column (75 μm diameter, 50 cm length) using 0.1% formic acid in water as solvent A and 0.1% formic acid in acetonitrile as solvent B. The samples were separated using a 180 min gradient from 8 to 30% solvent B at a flow rate of 300 nL/min. The survey MS scan was acquired in the range 350–1650 *m*/*z* with a resolution of 70,000 at *m*/*z* 200. The Q-exactive was operated in the data-dependent mode using a top 20 with an isolation width of 1.2 *m*/*z*. High-energy collisional dissociation (HCD) scans were acquired with a normalized collision of 32. Maximum ion injection time for the survey scan and MS/MS scan was 20 and 100 ms, respectively.

Raw MS/MS files were processed with Proteome Discoverer ver 2.4 (Thermo Fisher Scientific, Waltham, MA, USA) using the SEQUEST HT algorithms against the UP000005640 human reference proteome including isoform sequences from the uniport-KB database. The database search parameters were as follows: full enzyme digest using trypsin with up to two missed cleavages allowed; a precursor ion mass tolerance of 20 ppm; a fragment ion mass tolerance of 0.02 Da; dynamic modifications of 15.995 Da for methionine oxidation and 42.011 Da for protein *N*-term acetylation; and static modifications of 57.021 Da for carbamidomethylation on cysteine residues and 229.153 Da for TMT on any *N*-terminus. The co-isolation threshold was set to 50%. The reporter ion intensities for TMT-labels were corrected for isotopic impurities as provided by the manufacturer. Peptide and peptide spectrum matches were confirmed by Percolator based on a 1% false discovery rate (FDR). Confidence criteria were set to a 1% FDR at the peptide and protein lists used for downstream analysis. The mass spectrometry proteomics data have been deposited to the ProteomeXchange Consortium via the PRIDE [84] partner repository with the dataset identifier PXD030881.

### 4.6. Transcriptome Data Analysis

Processed transcriptome data and raw sequenced reads were downloaded from NCBI GEO (GSE174152 and GSE39870) and SRA database (SRR6493747-SRR6493750, SRR6493760-SRR6494762, SRR13398517-SRR13398519, SRR13398523-SRR13398525, SRR2017562, and SRR2017563), respectively. Downloaded sequenced reads were converted from the SRA file into FASTQ file format using fastq-dump (Version 2.8.0). To obtain high-quality reads from raw data, sequenced reads were pre-processed by Trimmomatic (Version 0.39, http://www.usadellab.org/cms/?page=trimmomatic, accessed on 5 January 2022) to remove low-quality sequences. The high-quality reads were aligned to the NCBI human reference genome (GRCh38) using Hisat2 (Version 2.1.0, https://daehwankimlab.github.io/hisat2, accessed on 5 January 2022) with the default parameters. The gene expression levels were calculated to FPKM using the cufflinks pipeline (Version 2.2.1, http://cole-trapnell-lab.github.io/cufflinks/, accessed on 5 January 2022). A differentially expressed gene was calculated between parental MCF-7 and anti-cancer-drug-resistant MCF-7 by the Cuffdiff program (Version 2.2.1) with default parameters. The publicly available transcriptome data and statistics were described in Table S4.

### 4.7. Statistical Analysis

R (Version 4.1) was used for all statistical analyses. Pair-wise comparison of proteome and publicly available microarray data between parental MCF-7 and anti-cancer-drug-resistant cell lines were performed using the *t*-test function in the stats package in R. The resulting *p*-values were processed to be adjusted *p*-values with the p.adjust function with the Benjamini–Hochberg method. 

### 4.8. Bioinformatics Analysis

The protein expression level was calculated from an abundance of mass spectrometry with the following normalization formula:

$$\begin{array}{c} \mathrm{Normalized}\;\mathrm{Abundance}\;\mathrm{intensity}\;\mathrm{of}\;\mathrm{protein}\;\mathrm{groups}\;=\;\mathrm{Abundance}\;\mathrm{Intensity}\;\mathrm{of}\\ \mathrm{Protein}\;\mathrm{Groups}/\mathrm{Sum}\;\mathrm{of}\;\mathrm{Abundance}\;\mathrm{Intensity}\;\mathrm{of}\;\mathrm{Samples}\;*\;1,000,000 \end{array}$$

The principal component analysis was performed using the *prcomp* function with global proteome expression profiles. Functional gene classification was performed with EnrichR based on the Gene Ontology database. The list of druggable proteomes was downloaded from ProteinAtlas (https://www.proteinatlas.org/humanproteome/tissue/druggable, accessed on 5 January 2022). The Kaplan–Meier survival analysis was performed on R with survival and survminer packages using TCGA-BRCA pan-cancer data and Metabric clinical data, which were downloaded by cgdsr packages. The hazard ratio was calculated by the univariant Cox proportional-hazards model on R with survival packages.

## 5. Conclusions

The comprehensive proteome and transcriptome analyses presented here revealed new insights on chemoresistance for three drugs. Our quantitative proteomics approach is a powerful method to target potentially valuable prognostic and therapeutic resistance biomarkers, enabling system-wide analysis and discovery of meaningful DEPs, leading to a better understanding of chemoresistance mechanisms. Along the way, our proteome study in drug-resistant breast cancer cells has identified several intriguing proteins that might be used as novel drug targets and prognostic biomarkers. Finally, our study highlights the two proteins as potential prognostic markers of chemotherapy resistance in breast cancer. 

## Figures and Tables

**Table 1:** Representative relative expression levels of protein between parental MCF-7 and drug-resistant cells.

Protein Name	MCF-7/DR		MCF-7/PR		MCF-7/TAR	
	Log_2_ FC	Adj *p*-Value	Log_2_ FC	Adj *p*-Value	Log_2_ FC	Adj *p*-Value
ATP6V1B2	0.2987	0.0007	0.1741	0.0167	0.4997	0.0034
MAP2K1	0.8756	0.0015	0.5689	0.0076	0.4647	0.0125
MAP4	0.7893	0.0005	0.2916	0.0218	0.3371	0.0141
PLIN3	0.8894	0.0007	0.6368	0.0057	0.2839	0.0189
PRKCI	0.1833	0.0195	0.2564	0.0060	0.6548	0.0060
ROCK2	0.2936	0.0131	0.2128	0.0198	0.1766	0.0322
SMN1	0.6062	0.0016	0.2697	0.0241	0.3360	0.0226
TXNRD1	0.8700	0.0007	0.3042	0.0137	0.6149	0.0121
FADS1	−0.2534	0.0226	−0.5072	0.0069	−0.8856	0.0137
FRK	−0.6422	0.0006	−0.2322	0.0043	−0.9870	0.0043
GAA	−0.6750	0.0010	−0.1442	0.0440	−0.3608	0.0133
HSD17B10	−0.6633	0.0054	−0.2812	0.0143	−0.3145	0.0308
CYP51A1	−1.0314	0.0072	−0.7026	0.0014	−0.6652	0.0209
DHODH	−0.9274	0.0051	−0.4792	0.0015	−0.1451	0.0329
CA12	−1.5826	0.0025	−0.7594	0.0020	−0.7171	0.0051
ACAA1	−1.3387	0.0002	−0.3037	0.0039	−0.3683	0.0282

**Table 2:** Summary of 12 prognostic marker candidates. The Log_2_ FC was calculated between parental MCF-7 and drug-resistant cells using TMT 6-plex data.

Protein Name	MCF-7/DR		MCF-7/PR		MCF-7/TAR	
	Log_2_ FC	Adj *p*-Value	Log_2_ FC	Adj *p*-Value	Log_2_ FC	Adj *p*-Value
ATP6V1A	0.3835	0.0084	0.3850	0.0082	0.4489	0.0181
VPS26B	0.7674	0.0013	0.6040	0.0014	0.4039	0.0040
PLXNB2	0.6396	0.0017	0.3654	0.0017	0.6469	0.0055
RNF214	1.2672	0.0005	0.1830	0.0081	0.3928	0.0114
THYN1	0.6490	0.0006	0.2734	0.0438	0.2793	0.0043
RIDA	−0.5185	0.0025	−0.2993	0.0059	−0.1191	0.0104
CPSF6	−0.4050	0.0085	−0.1964	0.0028	−0.2366	0.0139
ABCD3	−0.9008	0.0040	−0.2900	0.0055	−0.8820	0.0104
UHRF1	−0.1969	0.0021	−0.1794	0.0328	−0.4596	0.0088
HNRNPL	−0.2741	0.0220	−0.1738	0.0414	−0.2100	0.0353
P4HB	−1.0898	0.0050	−0.3169	0.0336	−0.3561	0.0023
ACOX3	−1.5011	0.0069	−0.2912	0.0193	−0.5299	0.0098

## Data Availability

The mass spectrometry proteomics data have been deposited to the ProteomeXchange Consortium via the PRIDE [84] partner repository with the dataset identifier PXD030881.

## Supplementary Materials

The following supporting information can be downloaded https://www.mdpi.com/article/10.3390/molecules27061762/s1. Figure S1: Distribution of number of unique peptides per protein identification in TMT 6-plex and TMT 10-plex experiments; Figure S2: Violin plots of CV (coefficient of variance) between biological replications in each experimental set; Figure S3: Volcano plots of significantly altered proteins between parental MCF-7 and drug-resistant cells in each TMT 6-plex experiments; Figure S4: Direct comparison analysis between three drugs and combined analysis using TMT 6-plex and 10-plex data; Figure S5: Heatmap showing relative expression levels between MCF-7 and drug-resistant cells of each experimental set with log2 fold change; Figure S6: Overall survival plot with Kaplan–Meier model with highly significant genes of Figure 6a under Metabric chemotherapy patient data; Figure S7: Overall survival plot with Kaplan–Meier model with highly significant genes of Figure 6b under Metabric chemotherapy patient data; Figure S8: Representative MS/MS spectrum of LQLEQQVATGPALDNK peptide in PLXNB2; Figure S9: Representative MS/MS spectrum of SPGADLSLEK peptide in ACOX3; Figure S10: Expression patterns of PLXNB2 and ACOX3 in TMT 10-plex data; Table S1: Raw abundance from proteome discoverer pipeline; Table S2: Normalized protein expression level of TMT 6-plex and TMT 10-plex dataset; Table S3: Result of significantly differentially expressed proteins; Table S4: Analysis results of public RNA-seq data from GEO and SRA; Table S5: Result of gene ontology analysis from positively correlated DEPs and DEGs of public transcriptome data; Table S6: Result of gene ontology analysis from common and unique identified drug-resistant DEPs; Table S7: Identified potential drug target proteins with relative expression levels; Table S8: Overall survival rate of resistant cell lines commonly regulated genes with TCGA and Metabric.
