# Supplementary material for: Discovery of Proteins Responsible for Resistance to Three Chemotherapy Drugs in Breast Cancer Cells Using Proteomics and Bioinformatics Analysis
Source: Molecules. 2022 Mar 8;27(6):1762. doi: 10.3390/molecules27061762 (PMC8954867; doi:10.3390/molecules27061762)
Supplement: Supplementary file 1 [file molecules-27-01762-s001.zip › supplementary_figures.pdf]

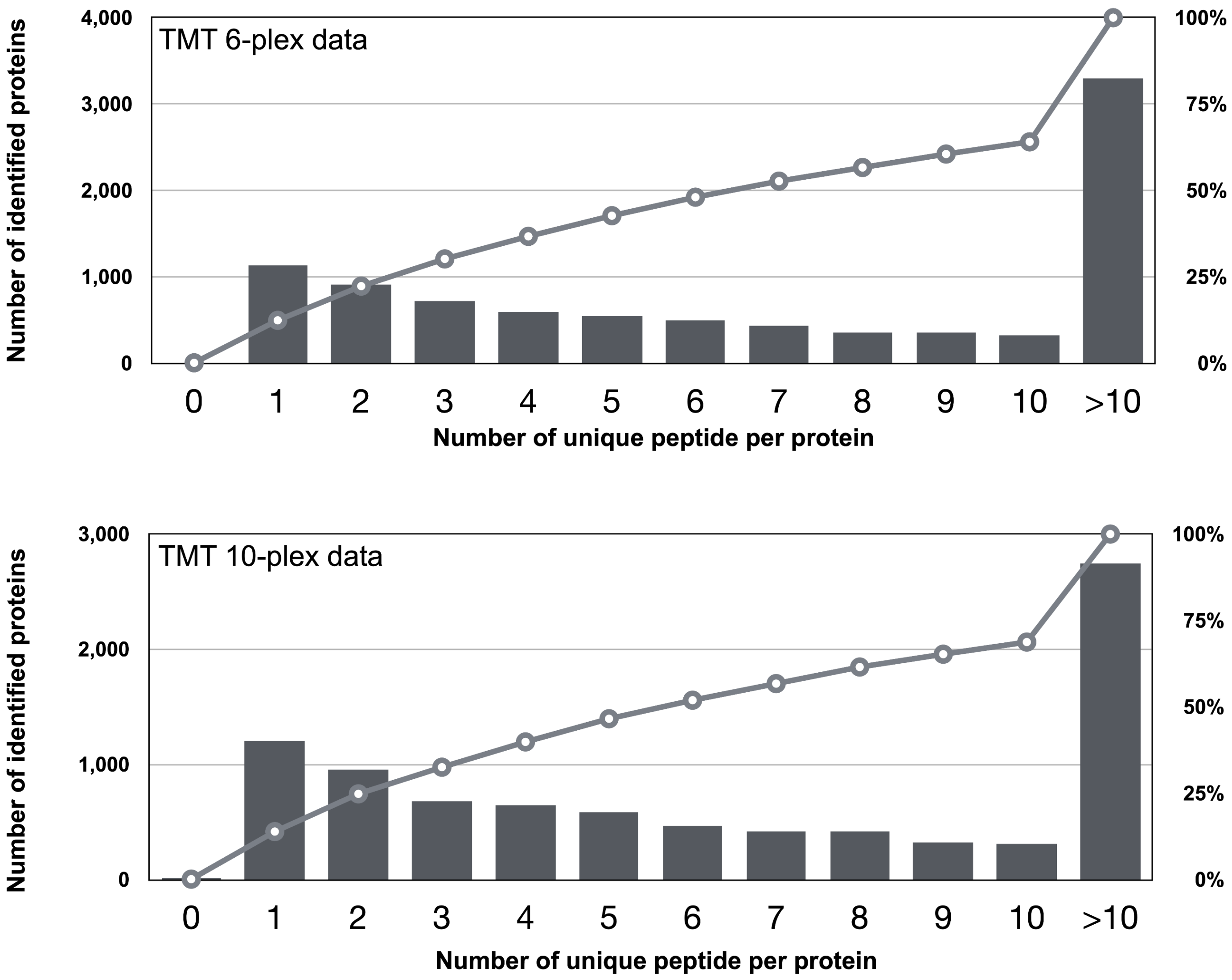

Figure S1. Distribution of number of unique peptides per protein identification in TMT 6-plex and TMT 10-plex experiments

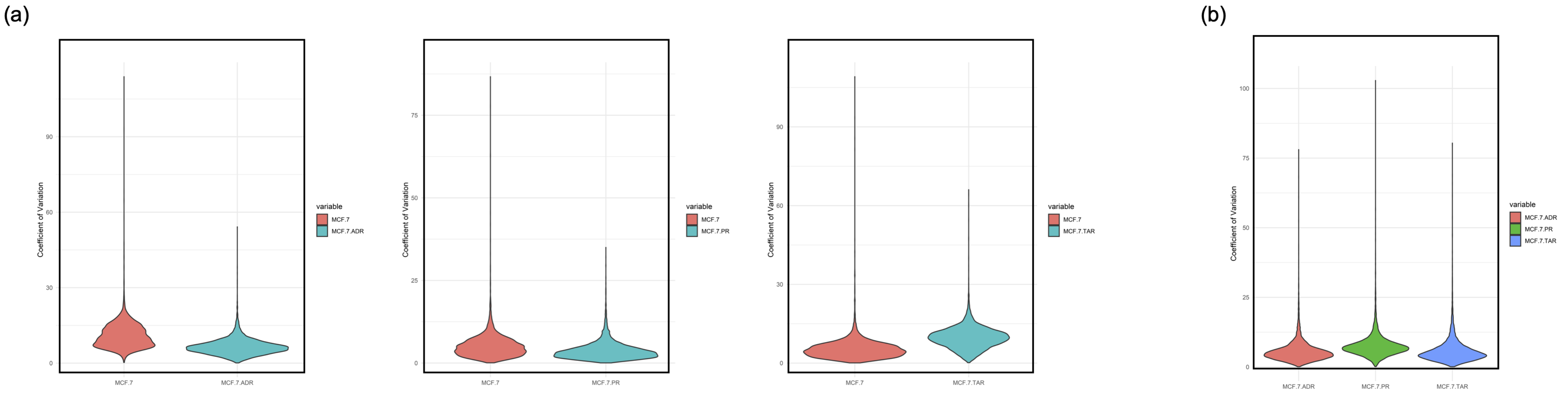

Figure S2. Violin plots of CV (coefficient of variance) between biological replications in each experimental sets. CV value calculate from the biological replicates of TMT 6-plex labels (a) and TMT 10-plex label (b) data

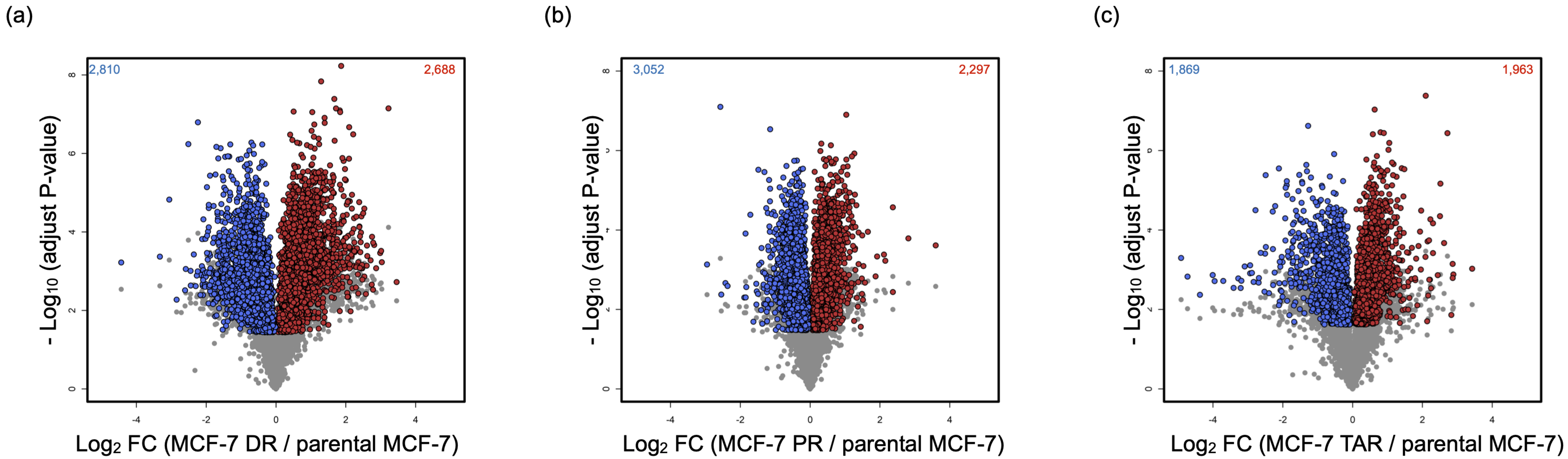

Figure S3. Volcano plots of significantly altered proteins between parental MCF-7 and drug-resistant cells in each TMT 6-plex experiments. The red and blue spots indicate significantly over-expressed proteins in drug-resistant MCF-7 cells (red) or parental MCF-7 (blue).

(a) (b)

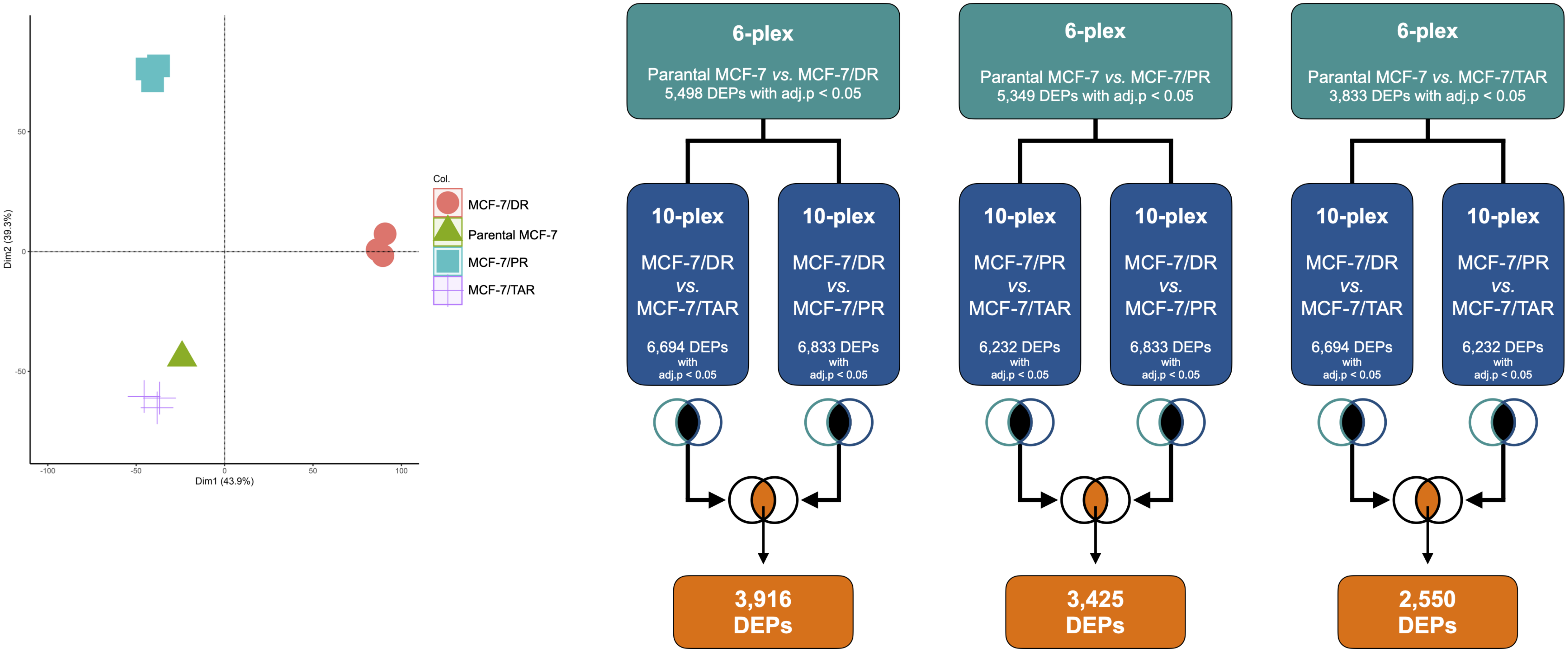

Figure S4. Direct comparison analysis between three drugs and combined analysis using TMT 6-plex and 10-plex data.
(a) Principle component analysis of proteins quantified in TMT 10-plex experiment. (b) Schematic diagram of combining process of DEPs between 6-plex and 10-plex data.

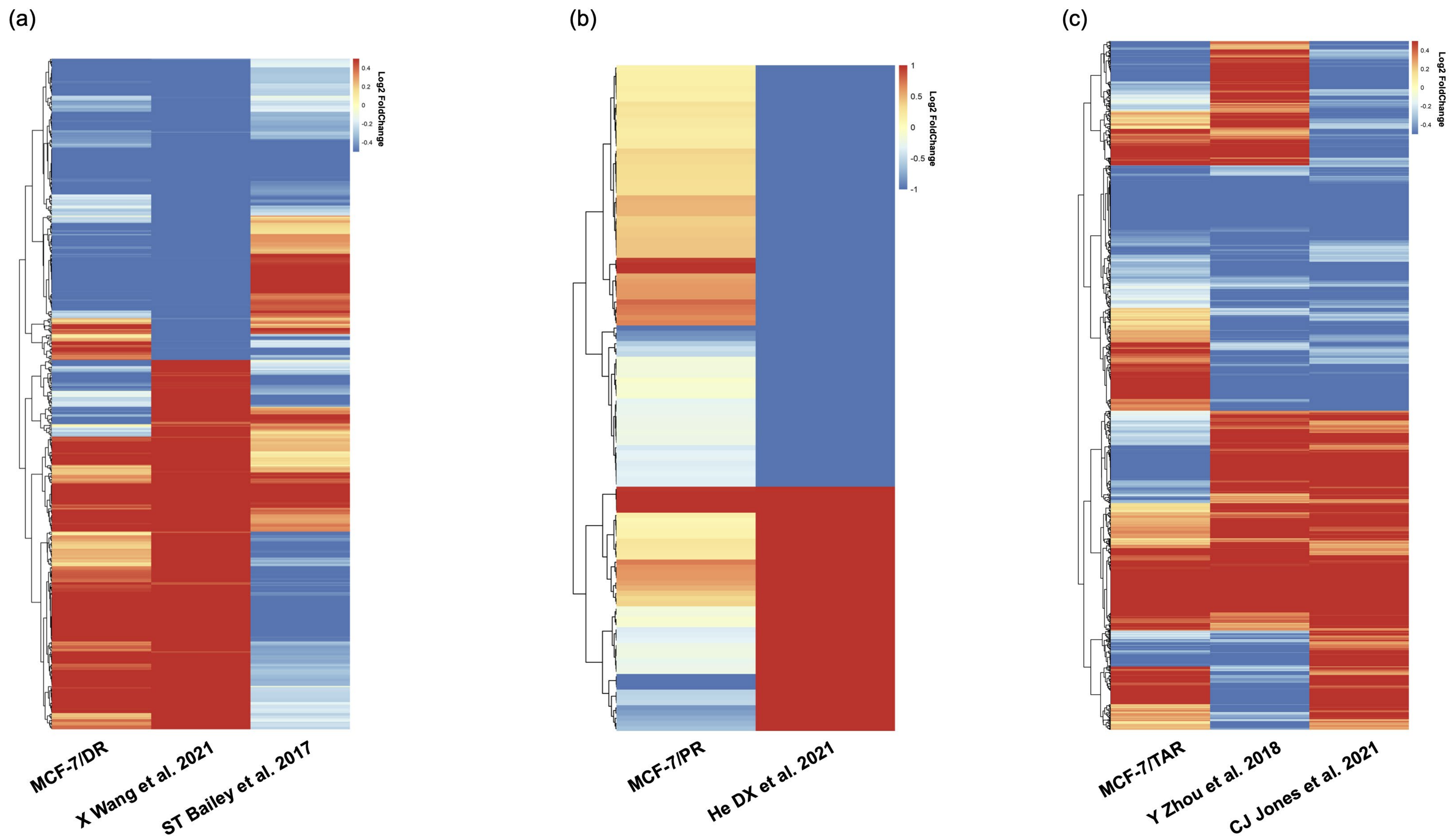

Figure S5. Heatmap showed relative expression levels between MCF-7 and drug-resistant cells in each experimental sets with log2 fold change.
(a) Expression patterns of 543 genes have been detected in three datasets of doxorubicin resistant cells. (b) Relative expression levels of 979 genes related to MCF-7/PR were identified from DEPs and DEGs. (c) The 1,058 genes were overlapped in both proteome and transcriptome. The expression levels were indicated with relative expression Levels by log2(fold-change).

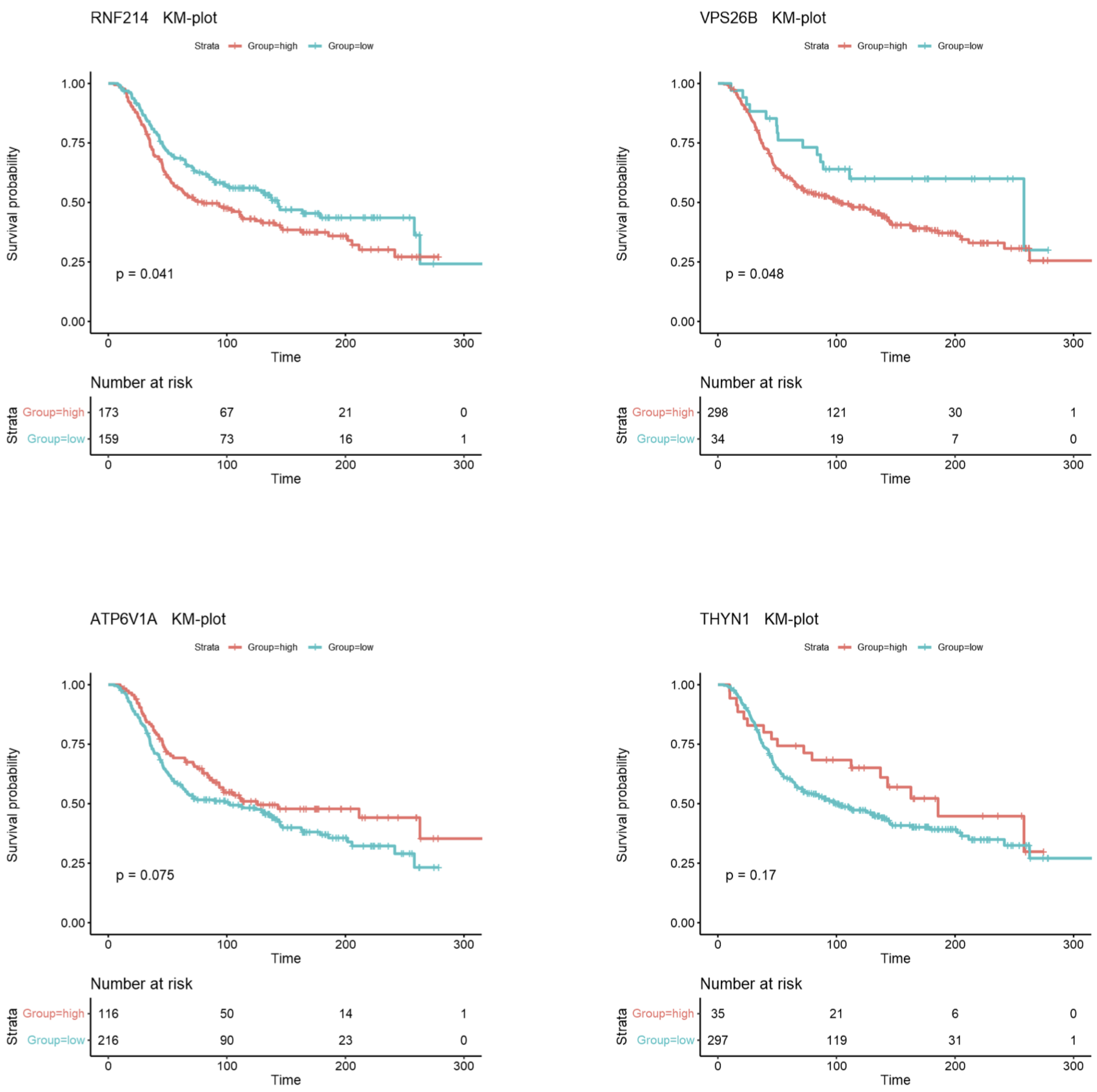

Figure S6. Overall survival plot with Kaplan-Meier model with highly significant genes of figure 6a under Metabric chemotherapy patients data

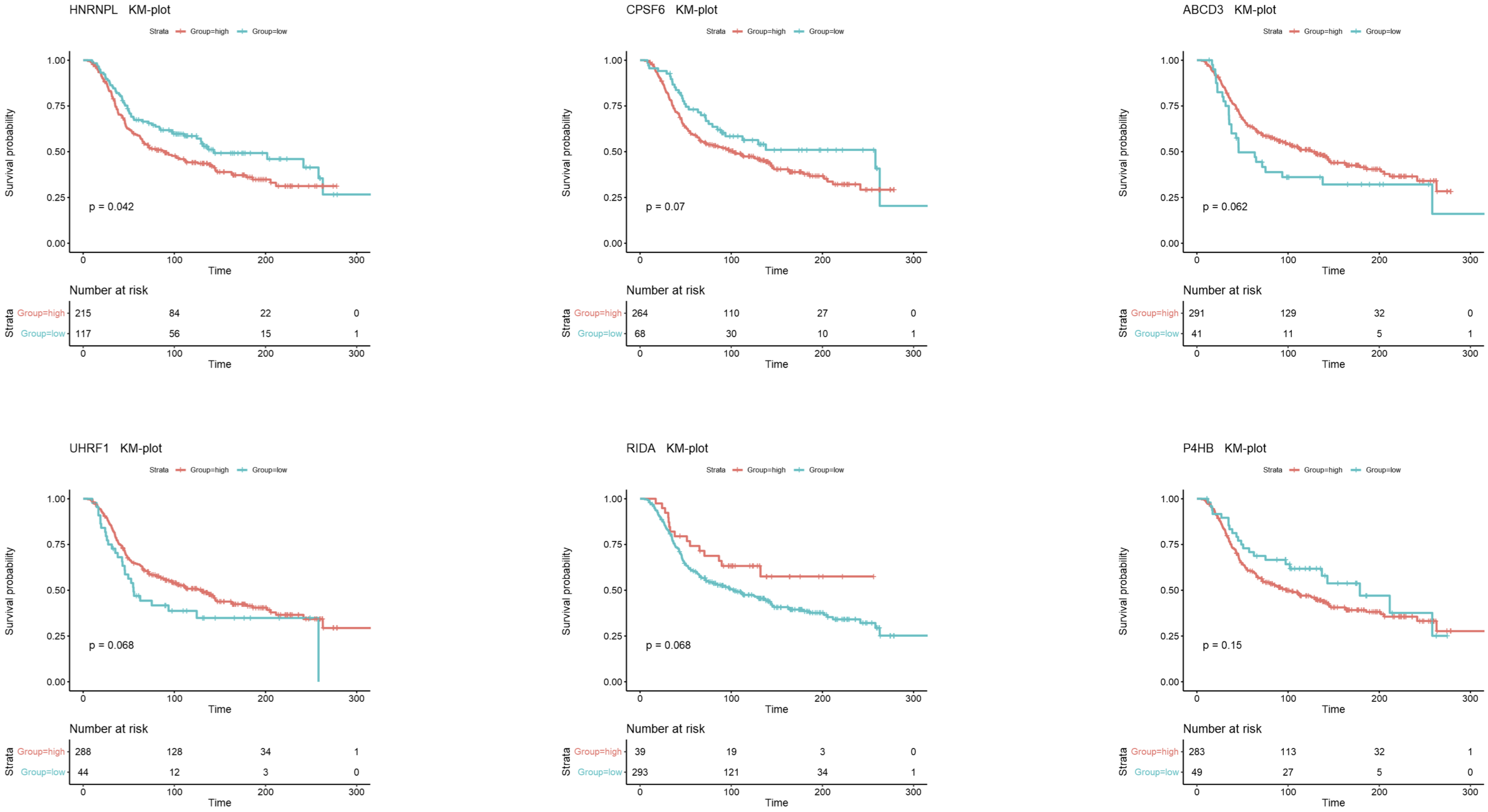

Figure S7. Overall survival plot with Kaplan-Meier model with highly significant genes of figure 6b under Metabric chemotherapy patients data

PLXNB2 (Score SEQUEST HT: 336.21)

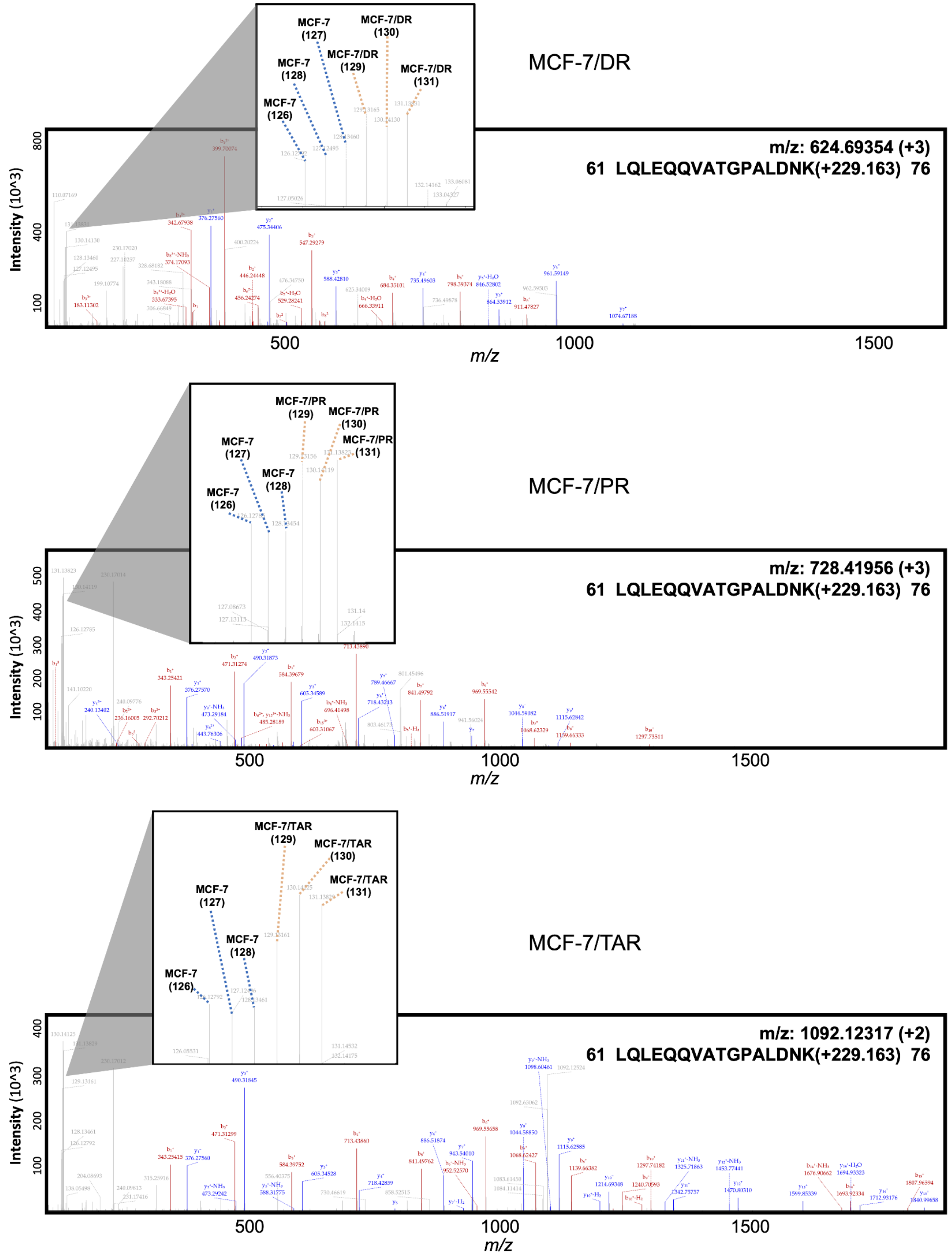

Figure S8. Representative MS/MS spectrum of LQLEQQVATGPALDNK peptide in PLXNB2

ACOX3 (Score SEQUEST HT: 414.44)

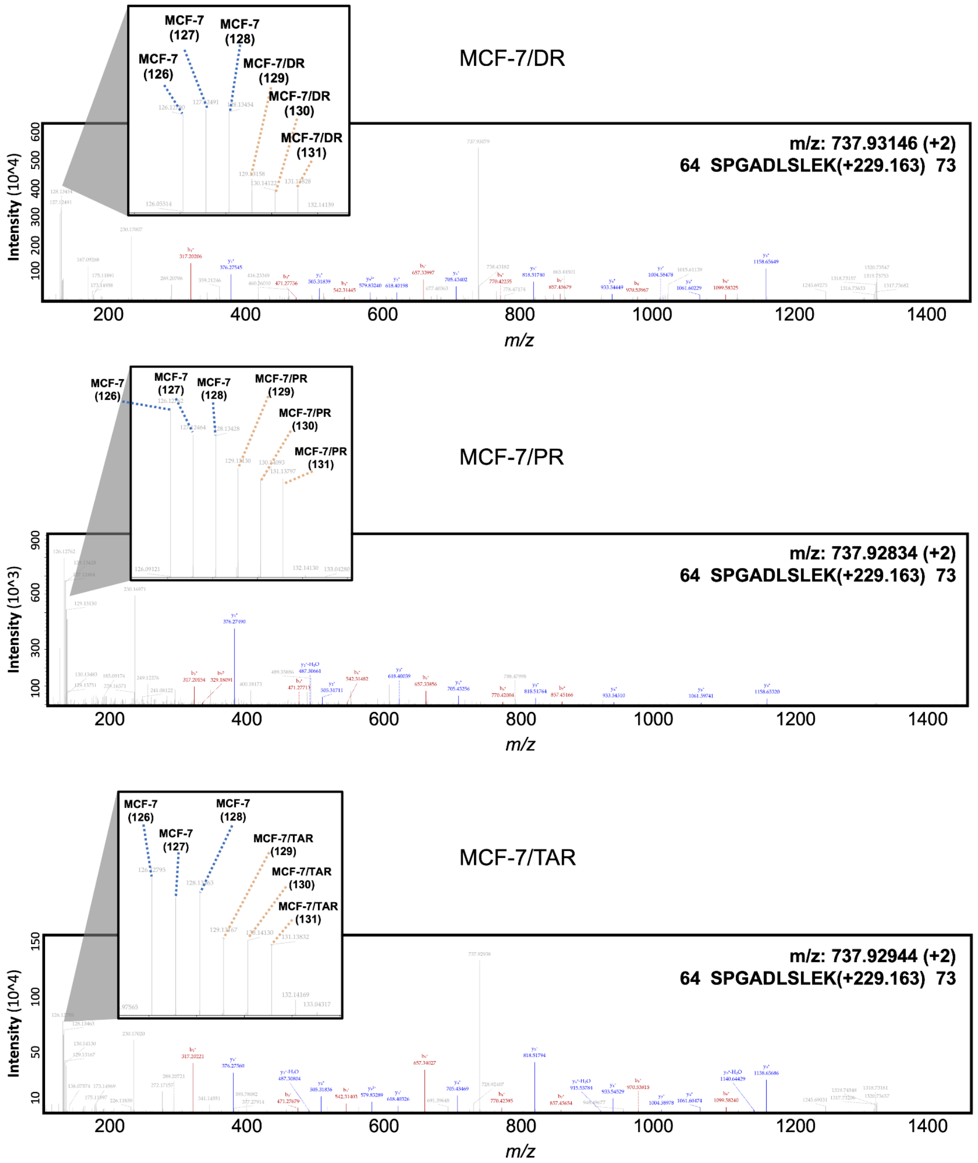

Figure S9. Representative MS/MS spectrum of SPGADLSLEK peptide in ACOX3

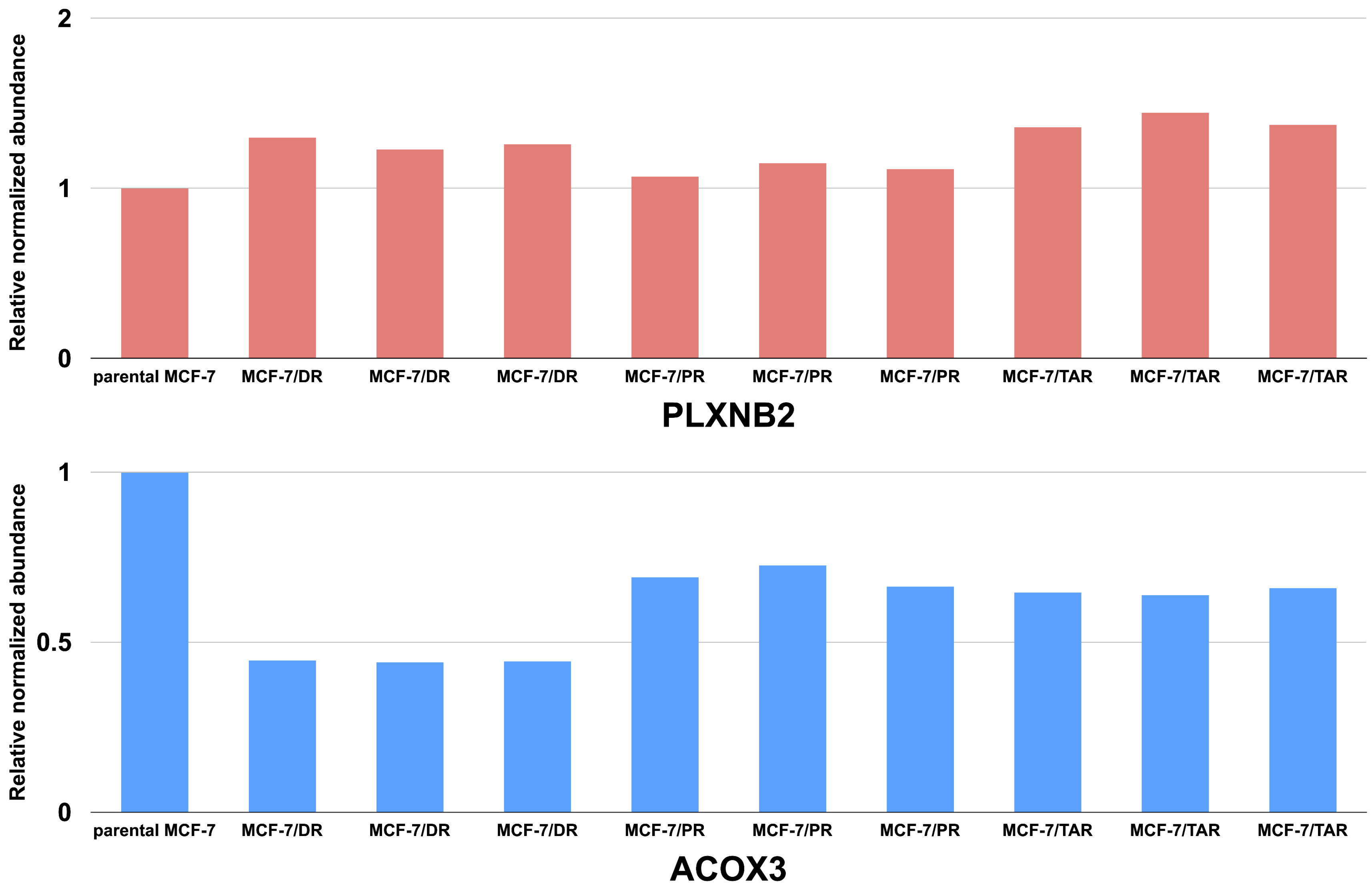

Figure S10. Expression patterns of PLXNB2 and ACOX3 in TMT 10-plex data
